# Tonsil biopsy to detect chronic wasting disease in white-tailed deer (*Odocoileus virginianus*) by immunohistochemistry

**DOI:** 10.1371/journal.pone.0282356

**Published:** 2023-03-30

**Authors:** David A. Schneider, Aaron D. Lehmkuhl, Terry R. Spraker, Robert O. Dittmar, Mitch A. Lockwood, Susan Rollo, Tracy A. Nichols

**Affiliations:** 1 United States Department of Agriculture, Animal Disease Research Unit, Agricultural Research Service, Pullman, Washington, United States of America; 2 Department of Veterinary Microbiology and Pathology, College of Veterinary Medicine, Washington State University, Pullman, Washington, United States of America; 3 United States Department of Agriculture, National Veterinary Services Laboratories, Animal Plant Health Inspection Agency, Ames, Iowa, United States of America; 4 Colorado State University Diagnostic Laboratory, College of Veterinary Medicine, Colorado State University, Fort Collins, Colorado, United States of America; 5 Texas Parks and Wildlife Department, Austin, Texas, United States of America; 6 Texas Animal Health Commission, Austin, Texas, United States of America; 7 United States Department of Agriculture, Cervid Health Program, Animal Plant Health Inspection Agency, Fort Collins, Colorado, United States of America; US Geological Survey, UNITED STATES

## Abstract

Chronic wasting disease (CWD) continues to spread in wild and farmed cervid populations. Early antemortem CWD testing of farmed cervids is of considerable interest to producers and regulatory agencies as a tool to combat this spread. The tissues accessible for antemortem sampling are limited and include biopsy of the tonsil and recto-anal mucosa-associated lymphoid tissue (RAMALT). The sensitivity to detect CWD by immunohistochemistry (IHC)—the regulatory gold standard—using biopsy samples of RAMALT from naturally infected white-tailed deer (WTD) has been determined by several studies. However, similar information is lacking for tonsil biopsy. In this study, two-bite tonsil biopsies from 79 naturally infected farmed WTD were used to determine the diagnostic sensitivity of tonsil IHC compared to the official CWD status based on results from the medial retropharyngeal lymph nodes and obex. IHC detection of CWD by tonsil biopsy was compared to the result and follicle metrics from the contralateral whole tonsil. The sensitivity of two-bite tonsil biopsy for detecting CWD by IHC was 72% overall. When the stage of infection was considered, the sensitivity was 92% for deer in late preclinical infection but only 55% for early preclinical infection. For deer with early preclinical infection, the sensitivity for deer homozygous for the prion protein gene (*PRNP*) coding for glycine at codon 96 (GG) was 66% but only 30% when heterozygous for the serine substitution (GS). The results indicate that the sensitivity of two-bite tonsil biopsy in WTD, and consequently its potential utility as an antemortem diagnostic, is limited during early infection, especially in WTD heterozygous for the serine substitution at *PRNP* codon 96.

## Introduction

Since its initial identification several decades ago, the incidence of chronic wasting disease (CWD) in North American wild and farmed cervid populations has increased. Since animals can transmit infection months to years before developing clinical signs, strategies to limit transmission depend on detecting affected stock during early infection. Diagnosis of CWD was initially limited to postmortem examination of clinical animals, where histologic analysis of the central nervous system would reveal characteristic spongiform neurodegeneration in advanced cases [1]. Antibody-based diagnostics were developed when the role of abnormally folded prion protein was recognized as central to prion diseases, accumulating in neural tissue before spongiform neurodegeneration and, in the case of cervids with CWD, even earlier in specific lymphoid tissues [2]. Immunohistochemistry (IHC) provides a great deal of diagnostic certainty. A sample is identified as positive when immunostaining specific for disease forms of the prion protein (e.g., PrP^CWD^) can be visualized in expected locations such as lymphoid follicles [3].

The official regulatory test of the United States Department of Agriculture (USDA) used for postmortem diagnosis of CWD is IHC of the medial retropharyngeal lymph nodes (MRPLNs) and the obex [4]. Though the MRPLNs are a site of early PrP^CWD^ accumulation in white-tailed deer (WTD) (*Odocoileus virginianus*) [5, 6], biopsy of the MRPLNs requires a surgical approach and is thus an impractical tissue source for routine antemortem diagnosis. Abnormal prion protein can also accumulate in the recto-anal mucosa-associated lymphoid tissue (RAMALT) in sheep, WTD, and elk, which is readily sampled through superficial mucosa biopsy in living animals [7–11]. However, the diagnostic sensitivity of IHC using RAMALT samples can vary between 25% and 95%, depending on animal species and genetic variability within the prion protein gene (*PRNP*) [10, 12–14]. Furthermore, IHC detection of PrP^CWD^ in the RAMALT of WTD can vary from 12 to 27 months after infection [15]. Accumulation of PrP^CWD^ in the palatine tonsils is a relatively early event in mule deer (*Odocoileus hemnionus*) and WTD [6, 16–19], and some antemortem tonsil biopsy data have been published [16, 20, 21]. From a retrospective study of WTD [16], PrP^CWD^ was detected in tonsil biopsies by IHC as early as six months post-inoculation. In the present study, we report the IHC diagnostic sensitivity of a two-bite tonsil biopsy [20] from 79 field cases in farmed WTD. All study deer were preclinical, and all samples, including tonsil biopsies, were collected postmortem. Also evaluated were the potential associations of infection stage, *PRNP* genotype at codon 96, and tonsil follicle metrics on detection of PrP^CWD^ by tonsil biopsy IHC.

## Materials and methods

### Sample collection

The study was carried out using tissues collected postmortem by employees of, and under the authority of, USDA-APHIS and Texas state regulatory agencies. These WTD herds were depopulated as an official regulatory action due to the presence of CWD in the herds. No animals were euthanized for the purpose of this study. All study samples were collected opportunistically postmortem.

All deer were considered preclinical and appeared healthy at the time of depopulation. Antemortem biopsy of the tonsil was performed in some of these animals as conducted by local regulatory agencies. Regulatory tissue samples (left and right MRPLNs, obex) were collected and submitted to the USDA National Veterinary Services Laboratories (NVSL) (Ames, IA) for official CWD IHC testing. After collecting the regulatory samples, a two-bite tonsil biopsy procedure was conducted as previously described [20], preserving the contralateral tonsil for unbiased metrics. In brief, the tongue was reflected and two biopsies were collected in situ from the left tonsil using a 6 mm ovarian biopsy instrument inserted into the left tonsillar crypt at a dorsolateral angle. When the tonsillar crypt was not large enough to insert the biopsy instrument, a bite of the overlying epithelium was first removed to expose the tonsil. The biopsies were placed into a tissue cassette with a sponge and put in 10% formalin. The biopsy technique mimicked the antemortem process as much as possible. To limit variation in the biopsy technique [20], all the samples were collected at diagnostic laboratories and a single operator (TAN) utilized the same procedure across depopulation groups. After tonsil biopsy, both whole tonsils were removed and placed in 10% formalin. The tonsil samples were held until the official CWD diagnostic reports were received from NVSL.

### Immunohistochemistry

Immunohistochemistry (IHC) was conducted at NVSL using the standard operating procedures for detecting PrP^CWD^ as previously described [6]. Briefly, 5 μm tissue sections were mounted on positively charged glass slides (Fisher Scientific), oven dried, treated with formic acid, rinsed with Tris buffer (pH 7.5), and subjected to hydrated autoclaving using DIVA antigen retrieval solution (Biocare Medical) and a decloaking chamber (Biocare Medical). Immunostaining was carried out using an automated immunostainer and associated reagents (Ventana Medical Systems) as well as the Anti-Prion (99) Research Kit, RTU (Ventana Medical Systems). The main reagents of these kits included decloaker solution, antibody block, monoclonal antibody F99, alkaline phosphatase-conjugated anti-mouse IgG secondary antibody, fast red chromogen, and hematoxylin. Each automated run included tissue controls from CWD-infected and non-infected deer.

### Data collection and statistical analyses

Age was either precisely known from records or only known at the birth year level. For quantitative purposes, ages were recoded into one-year age groups such that precise ages were rounded up if equal or greater than a one-half year. The genotypes of the prion protein gene (*PRNP*) at codon 96—coding for the amino acids glycine (G) and serine (S)—were determined by a commercial service (GeneCheck). Genotypes at other codons were not determined. The stage of preclinical infection was classified by IHC of both MRPLNs and the obex, where ‘early’ stage deer had PrP^CWD^ accumulation in MRPLN follicles but not the obex, and ‘late’ stage deer had accumulation at both tissue locations. The PrP^CWD^-positive and total numbers of lymphoid follicles were counted in a thin section of the whole tonsil. Data were analyzed and graphed using the procedures available in SAS 9.4 (SAS Institute Inc.). Basic statistics and histogram plots were produced using the UNIVARIATE procedure. The FREQ procedure was used to calculate diagnostic sensitivities, exact 95% confidence limits (CLs), and measures of agreement (Cohen’s kappa coefficient, κ; McNemar’s *Q* test for 2x2 contingencies, *Q*_*M*_; Cochran’s *Q* test for stratified contingencies, *Q*_*C*_). Values of κ were categorized as one of six agreement levels [22]: none = 0–0.20, minimum = 0.21–0.39, weak = 0.40–0.59, moderate = 0.60–0.79, strong = 0.80–0.90, almost perfect > 0.90. The LOGISTIC procedure was used to test the association of stage of infection with genotype, sex, and age group and included first and second-order effects. The GLIMMIX procedure was used to model the effects of genotype, sex, and age on the total follicle counts of the whole tonsil sample (distribution: negative binomial). The likelihood ratio (*Q*_*LR*_), 95% CLs, and fit plots were used to assess the significance of each regression model.

## Results

**Fig 1:**
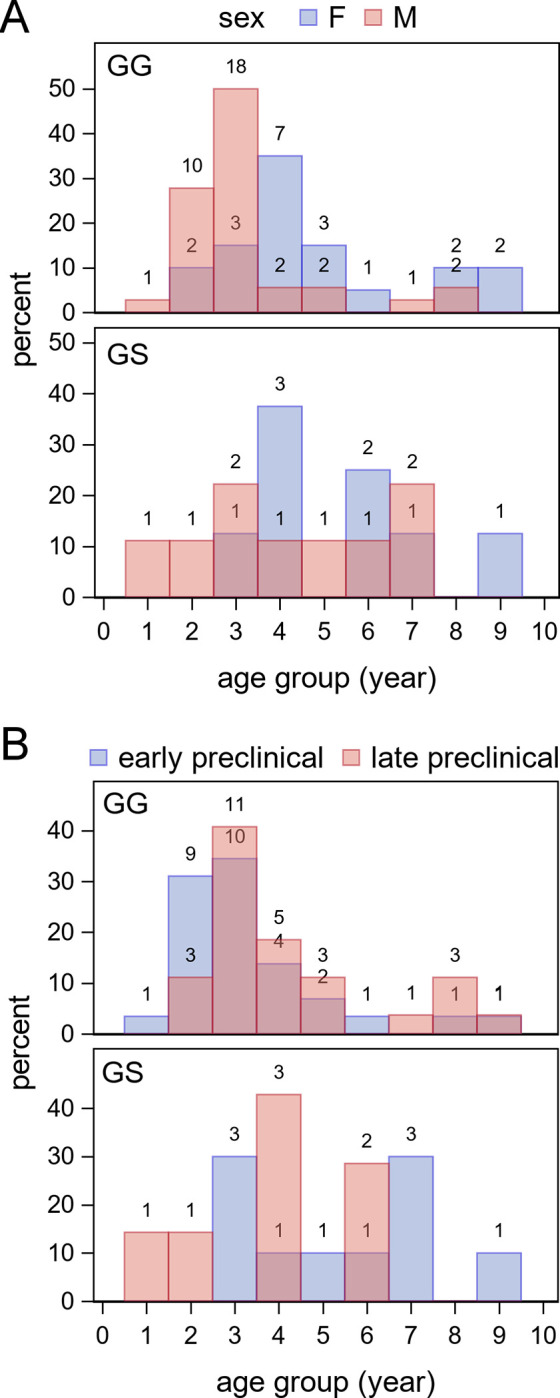
Distributions of ages by genotype at *PRNP* codon 96 grouped by sex (A) or stage of infection (B). The number of deer is indicated above each histogram bin.

Seventy-nine (31 female and 48 male) WTD deer from nine herds were identified as infected with CWD by official testing at NVSL. The data collected from these animals are provided in S1 Table. The dates of birth were known for 47 deer; the ages of 29 deer were recorded in whole years. Ages ranged from two deer less than 1 year of age to two deer aged 9.29 years (mean of original age data, 4.01 years). The median of ages grouped by year was 3 years. *PRNP* codon 96 genotypes included 56 GG, 17 GS, and one SS deer; genotype was not available for two female and three male deer. The age and sex distributions for genotypes GG and GS are shown in Fig 1A; the single SS deer was a 6.33-year-old female. Accumulation of PrP^CWD^ in MRPLN follicles was observed in all 79 deer. The stage of infection was classified as early preclinical in 42 deer (25 male, 17 female) and late preclinical in 37 deer (23 male, 14 female). The age distributions of deer in early and late preclinical stages of infection are shown for *PRNP* codon 96 genotypes GG and GS in Fig 1B; the SS deer was in early preclinical infection. The probability of a deer being in an early stage of preclinical infection was not dependent on age group, *PRNP* genotype (where codon 96 was either GG or GS), sex, or any interaction of these factors (*Q*_*LR*_, *P* = 0.2037).

### Diagnostic sensitivity of tonsil IHC using sections of whole and biopsy sample types

Upon official diagnosis, the paired postmortem samples of whole tonsil and two-bite tonsil biopsy were submitted to NVSL for evaluation by IHC. Postmortem tonsil biopsies from all deer had more than six lymphoid follicles present. Tonsil biopsies collected antemortem were considered inconclusive if accumulation of PrP^CWD^ was not observed and fewer than six lymphoid follicles were present in thin sections. Accumulation of PrP^CWD^ in antemortem tonsil biopsies was detected in 14 of 36 WTD in which antemortem sampling had been conducted (S1 Table). Of the 22 WTD in which PrP^CWD^ was not detected antemortem, 12 were detected in a postmortem biopsy. Conversely, tonsil accumulation of PrP^CWD^ in antemortem biopsies was not detected in any WTD in which accumulation was not detected in the postmortem biopsy. Hereafter, all results are for postmortem samples.

Accumulation of PrP^CWD^ was observed in a thin section of whole tonsil in 69 deer (diagnostic sensitivity = 87.3%) and in the tonsil biopsy samples of 57 deer (72.2%). Accumulation of PrP^CWD^ in a tonsil biopsy was only observed when accumulation was also observed in the whole tonsil. These paired estimates of general diagnostic sensitivity (i.e., without consideration of other factors) were significantly different (*Q*_*M*_ = 12.0, *P*_*exact*_ = 0.0005). Furthermore, the agreement of diagnoses between sample types was categorized as weak (κ = 0.5460, 95% CLs: 0.3352, 0.7568) but was better than by chance alone (*P*_*exact*_ < 0.0001). The following analyses compare diagnostic sensitivities (Table 1) and agreement as stratified by stage of preclinical infection and by genotype at *PRNP* codon 96.

**Table 1:** Immunohistochemical diagnostic sensitivities for paired tonsil sample types harvested postmortem from white-tailed deer with chronic wasting disease.

*PRNP* genotype	Early preclinical deer			Late preclinical deer			Overall		
	N	Whole	Biopsy	N	Whole	Biopsy	N	Whole	Biopsy
**GG**	29	90%	66%	27	93%	93%	56	91%	79%
								(80%, 97%)	(66%, 88%)
**GS**	10	60%	30%	7	86%	86%	17	71%	53%
								(44%, 90%)	(28%, 77%)
**SS**	1	100%	100%	0	-	-	1	100%	100%
**Unknown**	2	100%	0%	3	100%	100%	5	100%	60%
**Overall**	42	83%	55%	37	92%	92%	79	87%	72%
		(69%, 93%)	(39%, 70%)		(78%, 98%)	(78%, 98%)		(78%, 94%)	(61%, 82%)

The total number (N) of deer and the diagnostic sensitivities of each sample type (whole tissue section versus biopsy) are indicated at each intersection of infection stage (columns) and genotype at *PRNP* codon 96 (rows). The 95% confidence intervals for the ‘overall’ estimates are indicated parenthetically.

The agreement of results between sample types depended on the stage of preclinical infection (*Q*_*c*_ = 20.7170; *P* < 0.0001). From deer in late preclinical infection, there were no discordant pairs of results (that is, there was perfect agreement between sample types), yielding a joint tonsil IHC diagnostic sensitivity of 91.9% (exact 95% CLs: 78.1%, 98.3%). For deer in early preclinical infection, there was minimum agreement between tonsil sample types (κ = 0.3898, 95% CLs: 0.1587, 0.6210) but which was better than by chance alone (*P*_*exact*_ < 0.0019). The early preclinical diagnostic sensitivity of whole tonsil IHC was 83.3% (exact 95% CLs: 68.6%, 93.0%) and for tonsil biopsy IHC was 54.8% (exact 95% CLs: 38.7%, 70.2%); these estimates were significantly different (*Q*_*M*_ = 12.000, *P*_*exact*_ = 0.0005).

The agreement of results between sample types significantly depended on genotype (GG vs GS) stratified by stage of infection (*Q*_*c*_ = 21.3860; *P* < 0.0001). For deer in late preclinical infection, there were no discordant pairs of results for either genotype, yielding a joint estimate of tonsil IHC diagnostic sensitivity in GG deer of 92.6% (exact 95% CLs: 75.7%, 99.1%) and in GS deer of 85.7% (42.1%, 99.6%); a significant difference between these joint sensitivities was not detected (*Q*_*C*_ = 4.000, *P* = 0.1353). In contrast, during early preclinical infection there was minimum agreement of results between tonsil sample types when from GG deer (κ = 0.3596, 95% CLs: 0.0423, 0.6770) and when from GS deer (κ = 0.4444, 95% CLs: 0.0071, 0.8818). The agreement of results from early preclinical GG deer was significantly better than by chance alone (*P*_*exact*_ = 0.0328) but agreement from early preclinical GS deer was not (*P*_*exact*_ = 0.1667). For early preclinical GG deer, the tonsil IHC diagnostic sensitivity for whole samples was 89.7% (exact 95% CLs: 72.7%, 97.8%) but for tonsil biopsy samples was 65.5% (exact 95% CLs: 45.7%, 82.1%); these estimates were significantly different (*Q*_*M*_ = 7.000, *P*_*exact*_ = 0.0156). For early preclinical GS deer, a statistical difference between tonsil IHC diagnostic sensitivity of whole samples (60.0%, 95% CLs: 26.2%, 87.8%) and tonsil biopsy (30.0%, 95% CLs: 6.7%, 65.3%) was not detected (*Q*_*M*_ = 3.000, *P*_*exact*_ = 0.2500).

### Relationship of whole tonsil metrics with the probability of detecting PrP^CWD^ in a tonsil biopsy

**Fig 2:**
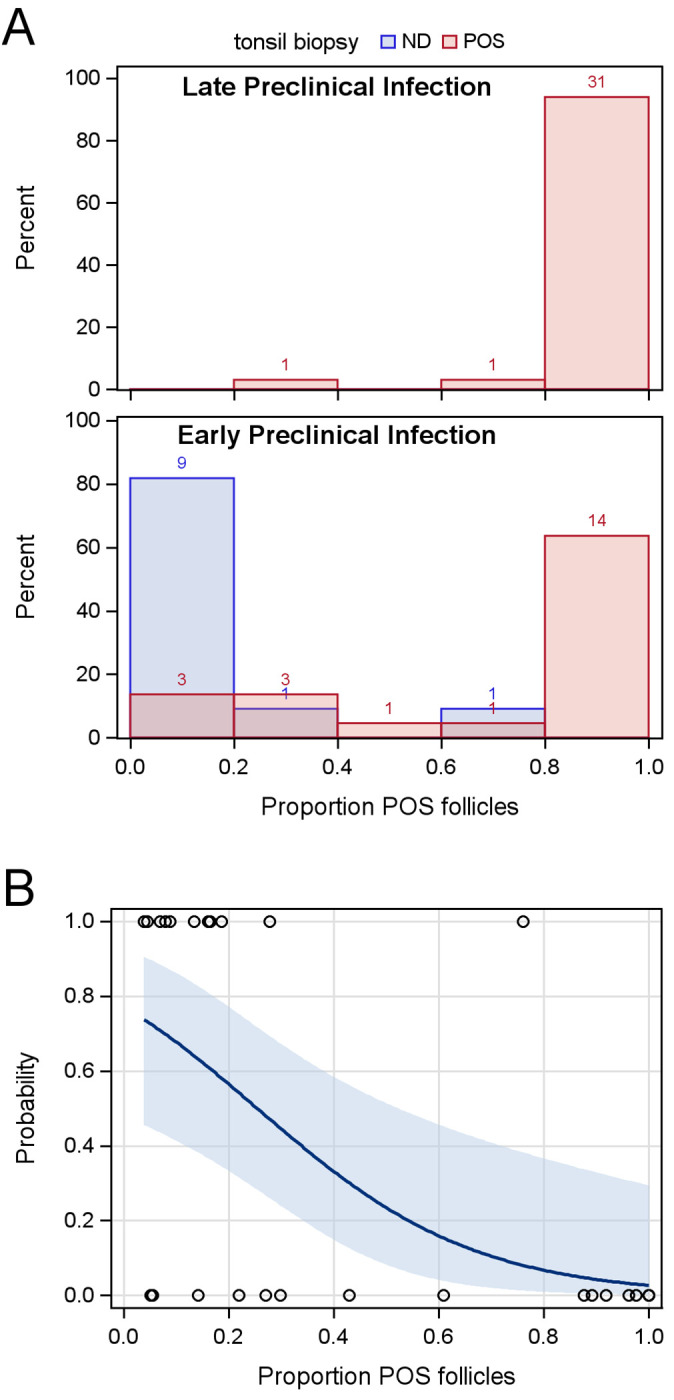
The relationship of tonsil biopsy results in white-tailed deer with estimates of the proportion of tonsil follicles positive for PrP^CWD^ accumulation. (A) The frequency (bar counts) of true positive (POS) and false negative (ND) tonsil biopsy results as stratified by stage of preclinical infection. (B) The predicted probabilities of a false negative tonsil biopsy result in white-tailed deer with early preclinical infection as a function of the estimated proportion of tonsil follicles positive for PrP^CWD^. The confidence band signifies the 95% confidence limits.

The proportion of PrP^CWD^ positive tonsil follicles was estimated by counting the total and positive numbers of follicles present in thin sections of the unbiopsied whole tonsil (N = 66 WTD; S1 Table). The total number of whole tonsil follicles counted was highly variable between deer (mean = 126.7, standard deviation = 47.5). The mean of whole tonsil follicle counts was marginally dependent on the animal’s age (*F* = 4.83, *P* = 0.0316); the estimated reduction in mean total follicle count was 4.9 follicles per year. The probability of a false negative tonsil biopsy result in early preclinical deer was not significantly dependent on the whole tonsil total follicle count (*Q*_*LR*_ = 0.3717, *P* = 0.5421). In contrast, the probability of a false negative tonsil biopsy result in early preclinical deer was significantly dependent on the whole tonsil estimate of the proportion of positive follicles (*Q*_*LR*_ = 30.4393, *P* < 0.0001; Fig 2A). The odds of a false negative result based on a two-bite tonsil biopsy from deer in early preclinical infection increased 1.617 (95% CLs: 1.226, 2.603) for each 0.1 unit decrease in positive proportion of whole tonsil follicles (Fig 2B).

## Discussion

Early detection of CWD-infected cervids is key to mitigating the spread of disease. Of particular interest is the potential application of antemortem diagnostic testing to farmed WTD. This study determined the sensitivity of CWD IHC using a two-bite biopsy technique reported to produce optimal antemortem retrieval of tonsillar follicles from white-tailed deer under field conditions [20]. In this study, diagnostically adequate numbers of follicles were obtained using this two-bite biopsy sampling technique in 79 preclinical, naturally infected farmed WTD from nine CWD-positive herds from across the United States. The study group included similar proportions of deer with early and late preclinical infections, and each infection stage was similarly represented by males and females, ages ranging from 6 months to 9 years, and the GG and GS genotypes of *PRNP* codon 96. The contralateral tonsil was collected intact to provide unbiased whole tonsil metrics to better understand factors that may affect the diagnostic sensitivity of tonsillar biopsy. The overall preclinical diagnostic sensitivity of CWD IHC using this unilateral two-bite tonsil biopsy technique was estimated to be 72% whereas the sensitivity from the paired whole tonsil was significantly higher at 87% (Table 1).

Animals with early preclinical infection—a stage defined in this study as official IHC detection of PrP^CWD^ in MRPLN follicles but not the obex of WTD—are notoriously difficult to diagnose antemortem. Thus, even though the diagnostic sensitivity of tonsil biopsy for WTD in late preclinical infection was 92% and was the same as that achieved by examining the whole tonsil, the tonsil biopsy sensitivity for WTD with early preclinical infection was reduced to 55% despite an 83% sensitivity based on the whole tonsil. Furthermore, early preclinical detection was low at 30% in WTD bearing the GS genotype of *PRNP* codon 96 as compared to 66% detection in GG herd mates.

The poor sensitivity of tonsil biopsy during early preclinical infection was strongly associated with the proportion of PrP^CWD^-positive tonsil follicles as estimated using follicle counts from the unbiopsied whole tonsil. As seen in Fig 2A, the detection of PrP^CWD^ in at least 80% of tonsillar follicles (x-axis) was observed in 31 (or 94%, y-axis) of 33 late preclinical deer and in 14 (42%) of 33 early preclinical deer. Thus, it is not surprising that PrP^CWD^ was detected by tonsil biopsy in all 45 of these deer. But false negative results from tonsil biopsies occurred when tonsil estimates fell below 80% positive follicles. In WTD with early preclinical infection and PrP^CWD^ present in the whole tonsil sample (N = 33), PrP^CWD^ was not detected by tonsil biopsy in two deer with respective estimates of 76% and 28% positive tonsil follicles, and in 9 of 12 (75%) deer in which the tonsil estimates were less than 20% positive follicles (range 4% to 19%). The odds of a false negative biopsy result during early preclinical infection increased by approximately 1.6 for every 10% decrease in the estimated proportion of positive tonsil follicles. As such, the chance of a false negative result from a unilateral two-bite tonsil biopsy was greater than 50% (probability 0.5) when the tonsils of WTD with early preclinical infection were estimated to have 20% or fewer positive follicles.

Other sample types and novel detection methods have been studied in naturally infected WTD as potential antemortem tests for CWD and, in each case, diagnostic sensitivity was negatively impacted by the *PRNP* genotype at codon 96 and for deer during the early stage of infection (all using the same definition as in this study). Deer with GS and SS codon 96 polymorphisms are still susceptible to CWD. However, the amount of PrP^CWD^ staining is significantly less in these animals as demonstrated in a controlled intranasal inoculation [6]. From a meta-analysis of IHC-based diagnosis using RAMALT biopsy [10], the sensitivity was 68% overall but was only 42% in GS deer and only 36% for deer in early preclinical infection. Newer assay methods detect the misfolding activity associated with prions and have the potential to detect far lower amounts of PrP^CWD^ than are routinely detected by immunoassays, including IHC. In one application of the real-time quaking-induced conversion (RT-QuIC) assay [23], the sensitivity of this misfolding assay applied to RAMALT biopsies was 69% overall but only 39% in GS deer and only 25% for deer in early preclinical infection. When the protein misfolding cyclic amplification (PMCA) assay was optimized for use with cervid blood samples [24, 25], the sensitivity was 79% overall but only 57% in GS deer and 53% for deer in early preclinical infection.

## Conclusions

While this study demonstrates some potential for using CWD IHC and tonsil biopsy as an antemortem diagnostic in naturally infected farmed WTD, detection was limited during early preclinical infection and in deer bearing the GS genotype at *PRNP* codon 96. This is not surprising given these same factors have been observed to negatively impact the measured diagnostic sensitivity of other antemortem sample types (e.g., RAMALT and blood), even when tested using protein misfolding assays. Thus, evaluations of CWD IHC applied to a two-bite biopsy sample of the palatine tonsil must also consider the potential impact of these limitations on its intended application.

## Supporting information

S1 Table Whole tonsil and tonsil biopsy results for seventy-nine preclinical white-tail deer naturally infected with chronic wasting disease. (XLSX)
